# FISHGLOB_data: an integrated dataset of fish biodiversity sampled with scientific bottom-trawl surveys

**DOI:** 10.1038/s41597-023-02866-w

**Published:** 2024-01-04

**Authors:** Aurore A. Maureaud, Juliano Palacios-Abrantes, Zoë Kitchel, Laura Mannocci, Malin L. Pinsky, Alexa Fredston, Esther Beukhof, Daniel L. Forrest, Romain Frelat, Maria L. D. Palomares, Laurene Pecuchet, James T. Thorson, P. Daniël van Denderen, Bastien Mérigot

**Affiliations:** 1 Center for Biodiversity & Global Change, Yale University, New Haven, CT USA; 2 Department of Ecology & Evolutionary Biology, Yale University, New Haven, CT USA; 3 Department of Ecology, Evolution & Natural Resources, Rutgers University, New Brunswick, NJ USA; 4 Changing Ocean Research Unit, Institute for the Oceans & Fisheries, The University of British Columbia, Vancouver, BC Canada; 5 FRB-CESAB, Montpellier, France; 6 MARBEC, Univ Montpellier, CNRS, IRD, IFREMER, Sète, France; 7 Department of Ecology & Evolutionary Biology, University of California Santa Cruz, Santa Cruz, CA USA; 8 Department of Ocean Sciences, University of California, Santa Cruz, Santa Cruz, CA USA; 9 National Institute of Aquatic Resources, Technical University of Denmark, Kongens Lyngby, Denmark; 10 Institute for Resources, Environment and Sustainability, The University of British Columbia, Vancouver, BC Canada; 11 International Livestock Research Institute, Nairobi, Kenya; 12 Sea Around Us, Institute for the Oceans and Fisheries, The University of British Columbia, Vancouver, BC Canada; 13 The Arctic University of Norway, Tromsø, Norway; 14 Alaska Fisheries Science Center, National Marine Fisheries Service (NOAA), Seattle, WA USA; 15 Graduate School of Oceanography, University of Rhode Island, Narragansett, RI 02882 USA

**Keywords:** Biogeography, Biodiversity, Community ecology, Ichthyology, Data integration

## Abstract

Scientific bottom-trawl surveys are ecological observation programs conducted along continental shelves and slopes of seas and oceans that sample marine communities associated with the seafloor. These surveys report taxa occurrence, abundance and/or weight in space and time, and contribute to fisheries management as well as population and biodiversity research. Bottom-trawl surveys are conducted all over the world and represent a unique opportunity to understand ocean biogeography, macroecology, and global change. However, combining these data together for cross-ecosystem analyses remains challenging. Here, we present an integrated dataset of 29 publicly available bottom-trawl surveys conducted in national waters of 18 countries that are standardized and pre-processed, covering a total of 2,170 sampled fish taxa and 216,548 hauls collected from 1963 to 2021. We describe the processing steps to create the dataset, flags, and standardization methods that we developed to assist users in conducting spatio-temporal analyses with stable regional survey footprints. The aim of this dataset is to support research, marine conservation, and management in the context of global change.

## Background & Summary

Spatio-temporal biodiversity observation programs—reporting taxa occurrence, abundance, biomass, and other characteristics—are important to monitor biodiversity, capture species distributions in space and time, and understand responses to global change. They are also used to estimate essential biodiversity variables and inform decision-making about conservation and management^1–3^. These programs vary widely in their sampling strategies, temporal and spatial extents, and taxonomic scope^4^. Differences in taxonomic, funding priorities, accessibility, and sampling possibilities have led to a heterogenous landscape of biodiversity observation programs, with some taxonomic groups and ecosystems more systematically sampled than others^5–7^. Nonetheless, long time-series play an outsized role informing basic science and public policy^8,9^ and represent unique opportunities to understand biodiversity and ecosystems under global change^10,11^. Sustaining such programs in the long-term requires substantial resources that are usually hard to maintain. Therefore, few monitoring programs regularly sample entire assemblages over large areas and long periods of time, and the existing data present an important opportunity for research and management.

Among spatio-temporal biodiversity observation programs, scientific bottom-trawl surveys (SBTS) represent a uniquely long-term and spatially extensive data source since their initiation in the 1960s. These surveys were initially designed to inform fish stock assessments with population abundance and weight estimations to determine sustainable fishing exploitation levels. SBTS are independent from commercial and recreational fishery catch data (i.e., they are fishery-independent data), and sample marine communities leaving close to the seafloor with a bottom trawl gear. On the contrary to fisheries trawling, SBTS follow protocols of sampling design to homogeneously sample entire regions and communities, and they operate under standardized and short sampling durations^12^. A global synthesis identified nearly 100 ongoing SBTS that target demersal marine communities living close to the seafloor on continental shelves and slopes around the world’s oceans^13^. SBTS are remarkable for their long time-series, regular sampling (typically at least once a year), their spatial coverage, and the diversity of the taxa sampled. Their strong link with management and fisheries has ensured SBTS relatively stable funding, with some surveys having been conducted continuously for more than 60 years (e.g., the North Sea and the Northeast US). Besides collecting crucial biological data to inform fish stock assessments, these surveys have proved valuable in understanding species co-occurrence and trophic relationships^14,15^, long-term community change^16^, biodiversity change and species on the move^17–19^, and community biomass changes^20^ within their survey regions.

Regional SBTS surveys are conducted all over the world, and therefore they can provide a unique opportunity for biogeographical and macroecological studies, particularly with regards to understanding full species range dynamics in space and time^13,21^ and for cross-ecosystem comparisons and syntheses^22^. These efforts require integrating multiple SBTS, which creates new research opportunities. Previous efforts to combine SBTS have informed our understanding of fishing impacts on sensitive species abundance trends^23^, changes in the trophic structure^24,25^, large-scale macroecological patterns of species and traits^26–28^, species range shifts^29^, community biodiversity and abundance dynamics^30,31^, and dynamics in thermal community composition in response to temperature changes^32,33^.

However, integrating large-scale survey datasets is a central challenge of ecological synthesis^34,35^. It is widely recognized that ecological datasets—each an irreplaceable snapshot of ecosystems in a unique place and time—are rarely collated, shared, and maintained in a format ideal for public use and reuse^34^. This occurs even when data are required to be shared publicly^36^ and arises partly due to the practical challenges of creating stable data repositories and making coding workflows fully reproducible^37^. Among ecological datasets, SBTS are hard to combine because of disparate expertise, differences in species catchability, lack of metadata, different data formats, different units of variables, different raw data processing available to users, and unequal sampling effort in time and space^13^. Some previous efforts have made survey data more available (https://datras.ices.dk/, https://oceanadapt.rutgers.edu/, https://apps-st.fisheries.noaa.gov/dismap/DisMAP.html), but SBTS have not yet been formally reconciled across continents, documented in a user-friendly format, and made readily available to the wider scientific community.

Solutions for moving toward better and more accessible ecological datasets include open science practices that foster computational reproducibility such as FAIR (Findable, Accessible, Interoperable, and Reproducible) software^38,39^, working in large and diverse teams where members bring unique knowledge and perspectives^35,40^, and publishing datasets that are designed for reuse and linked from major online clearinghouses^41^. Applying these solutions to synthesize SBTS datasets—a disparate collection of independent surveys that, if collated, would become one of the largest sources in marine ecology—would provide an invaluable service to the research community.

**Fig. 1:**
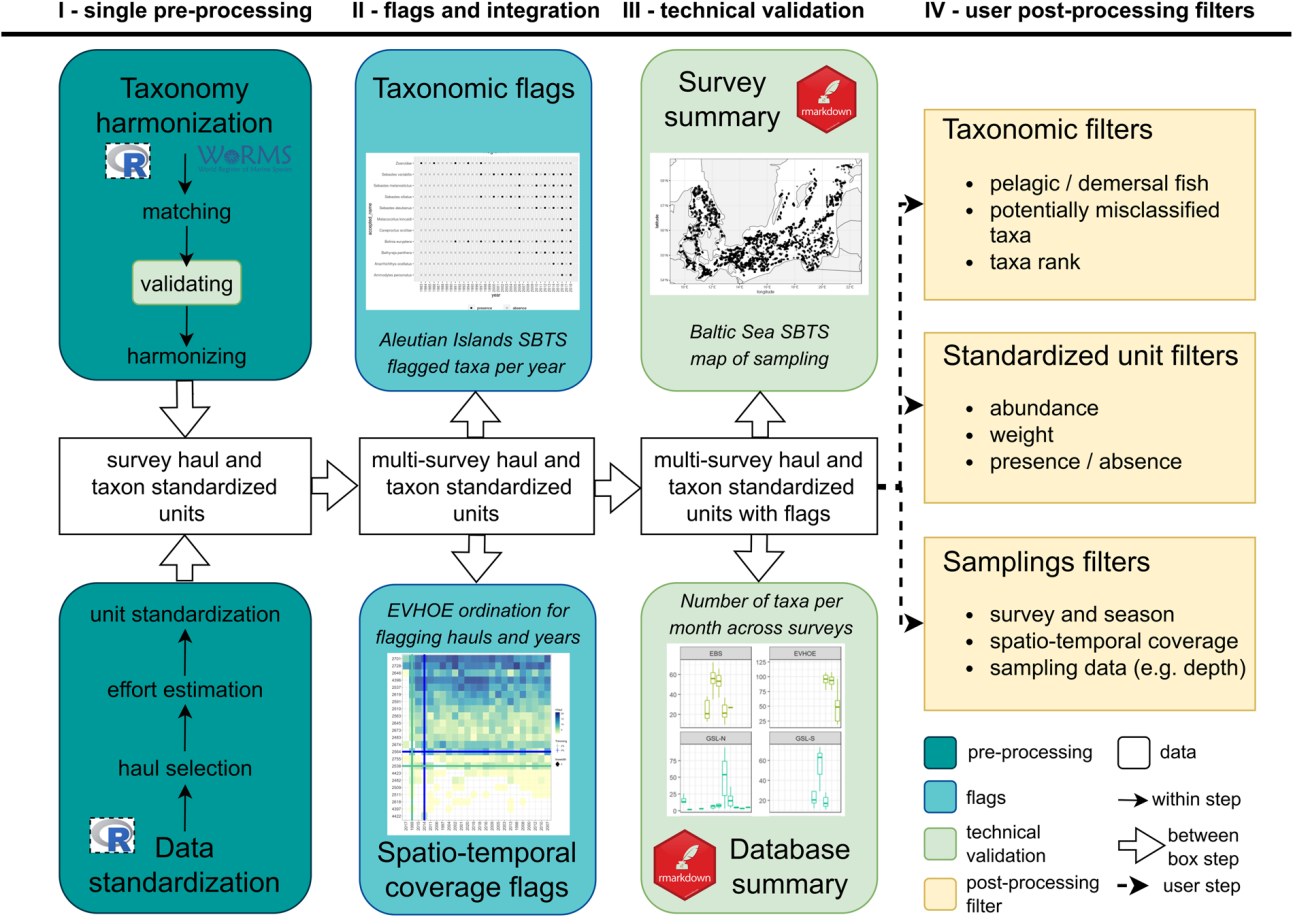
Conceptual diagram of FISHGLOB_data pre-processing, integration, flagging, and post-processing. (I) Single SBTS data pre-processing via taxonomic harmonization and data standardization; (II) Integration of taxonomic and spatio-temporal coverage flags; (III) Integration of STBS single surveys into one unique dataset, and investigation of survey and datasets summaries for technical validation; and (IV) Post-processing filters for end-users.

Here, we present FISHGLOB_data, a pre-processed fish community dataset compiling 29 regional SBTS from North American and European continental shelves and slopes. For transparency and reproducibility, we detail the methodological steps designed to develop this spatio-temporal dataset (Fig. 1). We used our previous experience and expertise with SBTS data to produce a dataset that can be used in many applications dealing with populations and community diversity or taxa presence-absence, abundance, and biomass. We present the extent of the dataset and provide guidance for end-users on use in research.

Compared to prior available resources mentioned above, FISHGLOB_data provides the first worldwide compilation of public SBTS data that crosses continents in a user-friendly format. With FISHGLOB_data, we facilitate the use of SBTS in the community by applying novel methods for standardizing the spatiotemporal footprint of each survey region and for identifying changes in species identification, which have been long-standing concerns and challenges in the analysis of SBTS and beyond.

By making use of our open-source workflow and codes, users can update the dataset with the most recent bottom-trawl surveys available, modify, adjust, and/or reproduce our processing methods, and provide feedback as relevant. This dataset complements the previous inventory of existing SBTS metadata^13^ by providing a user-friendly version of the publicly available survey data. Many other SBTS remain non-public. While the previously published SBTS metadata inventory improved findability and accessibility, the dataset introduced in this paper enhances general use, interoperability, and reproducibility, therefore making progress towards the FAIR principles^42^. More generally, this paper contributes to open science practices, particularly around methods and resources^34,43^ for the community of ecology and fishery scientists who would use the data in their research.

The methods and infrastructure presented here are general enough to integrate other bottom-trawl surveys that exist but may not be publicly available^13^. For example, the MEDITS bottom-trawl survey program (grouping northern Mediterranean SBTS conducted annually since 1994^44^) and the Icelandic bottom-trawl survey conducted since 2005^45^ are not publicly available. In efforts separate from this paper, these, and other surveys, have been integrated following the methodology outlined in this paper, after obtaining permission from data providers and regional experts and institutions. For many surveys that are not publicly available, metadata and contacts are provided in Maureaud *et al*.^13^. Establishing standardized data processing methods enables collaborations even when raw survey data are not publicly available, since the data may be accessible for specific research projects. A standardized methodology and network of users also help enhance the visibility of existing survey efforts, generate new scientific opportunities and potential collaborations, and enable knowledge transfer between scientific communities and countries.

## Methods

In this section we explain the methodological steps developed and applied to produce the FISHGLOB_data. First, we standardized and harmonized each single STBS to a unique format (Fig. 1, (I)). Second, we developed a flagging methodology per survey to inform potential additional taxonomic harmonization and ensure consistent spatio-temporal footprint per survey (Fig. 1, (II)). Third, we produced survey and cross-survey summaries used for technical validation of data processing (Fig. 1, (III)). Finally, we present user guidance (Fig. 1, (IV)) on how to conduct research using the dataset.

### Public survey data compilation

**Fig. 2:**
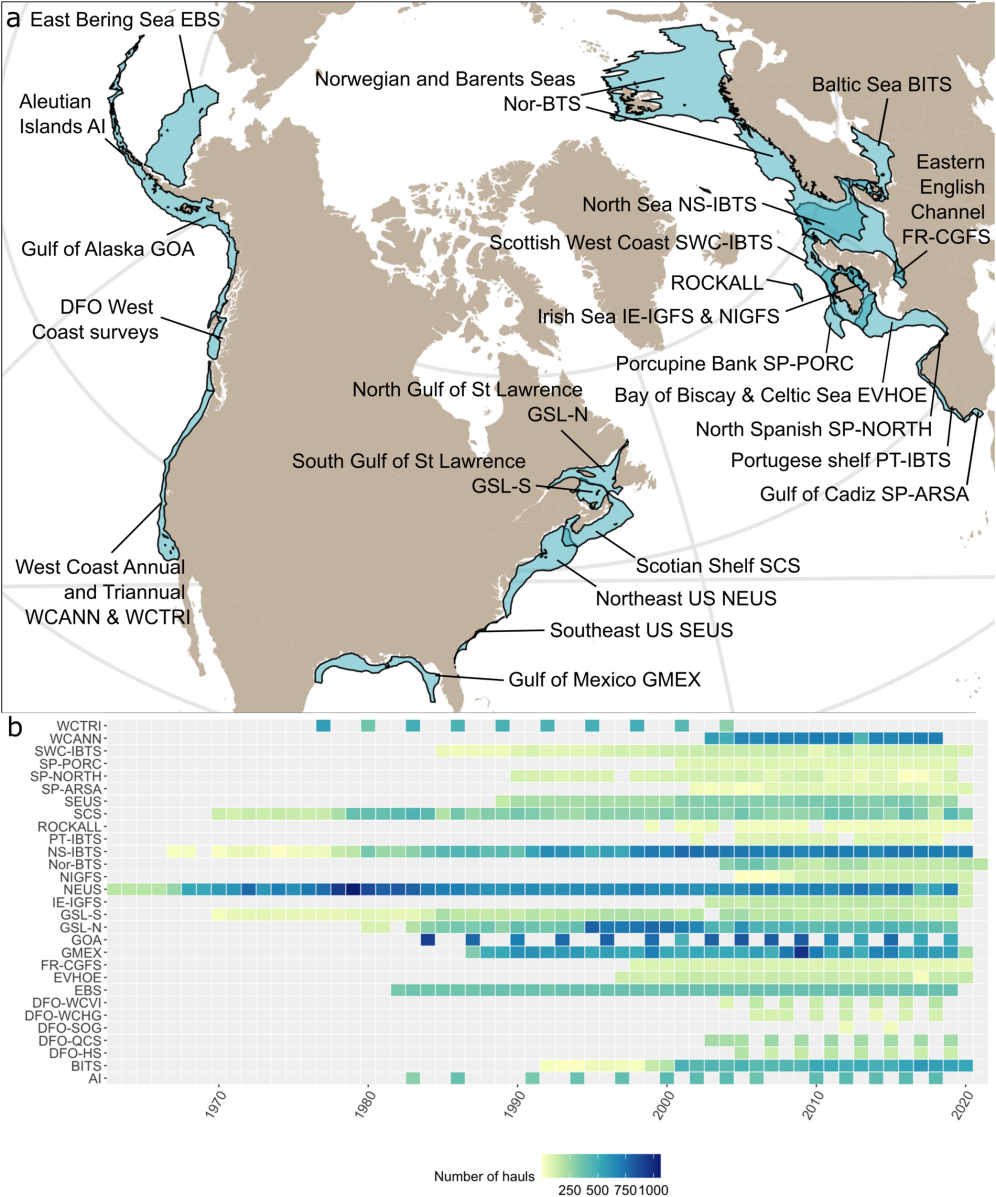
Spatio-temporal coverage of the FISHGLOB_data including 29 bottom-trawl surveys. (**a**) Survey spatial convex hull extracted from Maureaud *et al*.^13^ for publicly available surveys included in the dataset. Each survey is indicated with its name and survey code detailed in Supplementary Table S1. (**b**) Survey-specific sampling effort time-series.

SBTS are regional programs sampling marine demersal communities inhabiting continental shelves and slopes. Many of these surveys are now open access: European surveys are available through the International Council for the Exploration of the Sea (ICES) and the Institute of Marine Research (IMR), and North American surveys are available through the National Oceanic and Atmospheric Administration (NOAA) and Fisheries and Oceans Canada (DFO). Building on previous regional efforts integrating SBTS (https://oceanadapt.rutgers.edu/, https://apps-st.fisheries.noaa.gov/dismap/DisMAP.html, https://datras.ices.dk/)^26,28,29,33,46,47^, we compiled 29 open-access SBTS from 1963 to 2020 (Supplementary Table S1, Fig. 2) that sample demersal communities from subtropical to polar marine continental shelves and slopes of North America and Europe.

### Data standardization

Although SBTS sample similar ecological guilds (fish and macroinvertebrates associated with the seafloor), with similar sampling gears (i.e., bottom trawl nets), many differences also exist in data formats, reported variables, sampling methods, precise type of gear, season, and location of sampling, and other aspects. Here, we developed a standardization process leveraging knowledge and methods from SBTS experts to facilitate comparative ecological research across survey regions, while noting that researchers must be aware of remaining differences among surveys that cannot be easily addressed. We standardized each survey dataset separately at the haul level. A “haul” is a unique fishing event that is the unit of sampling; and a “station” is the location where the haul is performed. Every SBTS records bottom-trawl catches by haul. For each survey, we proceeded as follows: (i) we removed data that failed quality filters, or data not related to our focal variables; (ii) we estimated missing focal variables based on available information, when possible; (iii) we harmonized the taxonomy to a consistent nomenclature; and (iv) we converted values to standardized units. We explain the details of these steps (Fig. 1) in the sections below.

#### Quality filters

We removed all hauls that were not considered valid by each survey’s own quality control flags (e.g., hauls in which the net was marked as having malfunctioned). To be kept in the dataset, a haul needed to have precise geolocation, date, taxonomic identification, taxa abundance and/or weight, haul duration and/or swept area, and gear used. Haul duration and swept area are proxies for sampling effort in terms of sampling time and area covered by the bottom trawl. We only kept hauls sampled with otter-trawl gears and excluded beam trawl surveys from this dataset because they sample demersal communities with a different catchability and detectability^13,48^ (Supplementary Table S1). Beam trawl surveys target shallower parts of the continental shelf and are conducted at smaller scales than most otter trawl surveys. Compiling a beam trawl database would be an interesting future effort requiring significant inventory and consortium-building across regions. Additional QAQC steps are provided in the Technical Validation section below.

#### Focal variables

We focused on ensuring that essential information for population and community ecology research was available for all hauls. This information included sampling geolocation variables (latitude, longitude, depth, station number), sampling date (year, month, day), swept area (the sampled surface area), haul duration (the total sampling time), and survey catches for each sampled fish taxon (abundance and/or weight). We selected surveys that report all fish individuals sampled, excluding surveys that focused only on the most important commercial species. Definition and overview of these and other variables are detailed in Supplementary Table S2.

Some surveys only recorded taxon-specific catches in abundance (i.e., number of individuals) whereas others only in weight. We strived to have taxon abundance and biomass units for each survey by estimating missing variables when possible. For ICES surveys, we estimated weight per taxon per haul using length data from the raw survey data and length-weight relationship coefficients ‘a’ and ‘b’ sourced from FishBase^49^, using the equation *w* = *al*^*b*^, where w is the weight, and l the length. Abundance and length information for several NOAA-sourced surveys is not (publicly) available, therefore the dataset only contains taxa weight for those surveys.

#### Taxonomy

Taxonomic information in each survey was curated using the World Register of Marine Species (WoRMS), a widely-used and recognized marine taxonomic classification and catalog^50^. While WoRMS is integrating FishBase as a reference taxonomic backbone, we used WoRMS because some STBS already use WoRMS codes to report taxa. We only kept fish taxa sampled by the selected surveys because macroinvertebrates were not as comprehensively or consistently sampled across the surveys. This was done by selecting taxa included in the following taxonomic classes: Elasmobranchii, Holocephali, Chondrostei, Holostei, and Teleostei. For ICES surveys, the ‘AphiaID’ was provided for each taxon, so each corresponding scientific name and classification was retrieved with the “worms” R package^51^. For all other surveys, the scientific name was provided. Each name was then checked against the WoRMS taxonomic backbone from November 2023, and names were updated to their most current valid name and classification. This ensured that names given in the past that are not considered valid anymore were harmonized to recent names. We corrected for inconsistencies in taxonomic identifications that were known to the authors for some surveys. We also associated each scientific name with its valid ‘AphiaID’ and classification rank. The names and codes from the raw survey data were preserved in the dataset to ease future homogenization (column names preceded by ‘verbatim’)^52^. When a name did not match directly against the WoRMS taxonomic backbone, we used the fuzzy matching tool from WoRMS and manually selected and entered matches. Names that could not be matched were removed from the dataset. We additionally added the FishBase^49^ ‘SpecCode’ when it existed as a direct match to the scientific name. We used the last updated version of FishBase available via the “rfishbase” R package from 2019^53^. We added this link to FishBase to facilitate interoperability with this dataset that is widely used for trait-based ecology. WoRMS follows the taxonomic backbone from FishBase, so matching accepted scientific names may be enough to allow interoperability between SBTS and FishBase.

#### Standardized units

The raw data from each survey do not include the same metrics for abundance and do not all record the area swept by each haul. Haul duration and area swept are necessary for converting the raw abundances into standardized metrics comparable across surveys, such as the catch-per-unit-effort (CPUE) and the catch-per-unit-area (CPUA)^54^. To calculate CPUE and CPUA, weight and/or abundance were divided by haul duration and swept area, respectively. The swept area variable was directly available or already used to standardize abundance in several NOAA-sourced surveys, but that is not the case in ICES-, DFO-, or IMR-sourced surveys. To calculate the swept area when this variable was not directly reported, we used the sampling distance and gear opening when available, or alternatively the haul duration, vessel speed, and gear opening^54–56^. For some hauls, the swept area could not be calculated in a particular survey due to missing data. We then fitted a linear model with swept area as a response variable and haul data from this survey as potential predictor variables, using combinations of haul duration, sampling depth, sweep length, country, and sampling vessel. We predicted missing swept area values based on the survey-specific linear model including the above-mentioned available predictors and assigned predicted swept area to the hauls with missing values. All these models are provided in detail: ‘get_datras.R’ for ICES surveys. When the gear opening could not be obtained at the haul-level to calculate swept areas, we used a standardized gear opening communicated by survey experts. This was the case for some DFO-sourced SBTS. More survey-specific details about the source of standardized units are indicated in Supplementary Table S3.

In the final dataset named FISHGLOB_data, we provide the abundance and/or weight estimate(s), the abundance and/or weight standardized by the haul duration (CPUE), and the abundance and/or weight standardized by the swept area (CPUA). As noted above under *Focal variables*, not all variables are available for each survey and some variables are estimates using length-weight conversions rather than on-board measurements. We provide the *Focal variables* aggregated per taxa per haul in the final dataset, which is the level of aggregation that allows best integration across all survey regions.

### Optional standardization flagging

We created options for additional standardization of the data that users may implement depending on their intended use. We focused on two additional standardization procedures that are often needed, namely flagging (i) taxa that may not be consistently identified through time per survey; and (ii) locations and years that fall outside a spatial footprint that has been consistently sampled through time for each combination of survey and season available.

#### Temporal taxonomic flags

Despite the taxonomic standardization, additional taxonomic inconsistences may remain. For example, some surveys have changed the taxonomic level at which they record certain taxa, such as when improved field guides allowed identification of a taxon to the species level, rather than the genus level in previous years. For known cases, we updated the taxonomic classification to be consistent through time, such as by moving species in a particular group to the genus rank. However, additional inconsistencies may remain. Therefore, we analyzed the presence of taxa per year and survey to identify other taxa that may require additional attention. A taxon was flagged when it was present in less than 95% of the years sampled and if taxa shifted from present to absent and vice-versa less than four times over the time-series. This method was performed on the ‘accepted_ name’ which already incorporated the taxonomic harmonization on ‘verbatim_name’ (Supplementary Table S2). These criteria were intended to detect species that may have changed naming convention but were not picked up during the taxonomic harmonization. Depending on the intended use, users may need to verify these records with experts familiar with the specific SBTS.

#### Spatio-temporal sampling flags

**Fig. 3:**
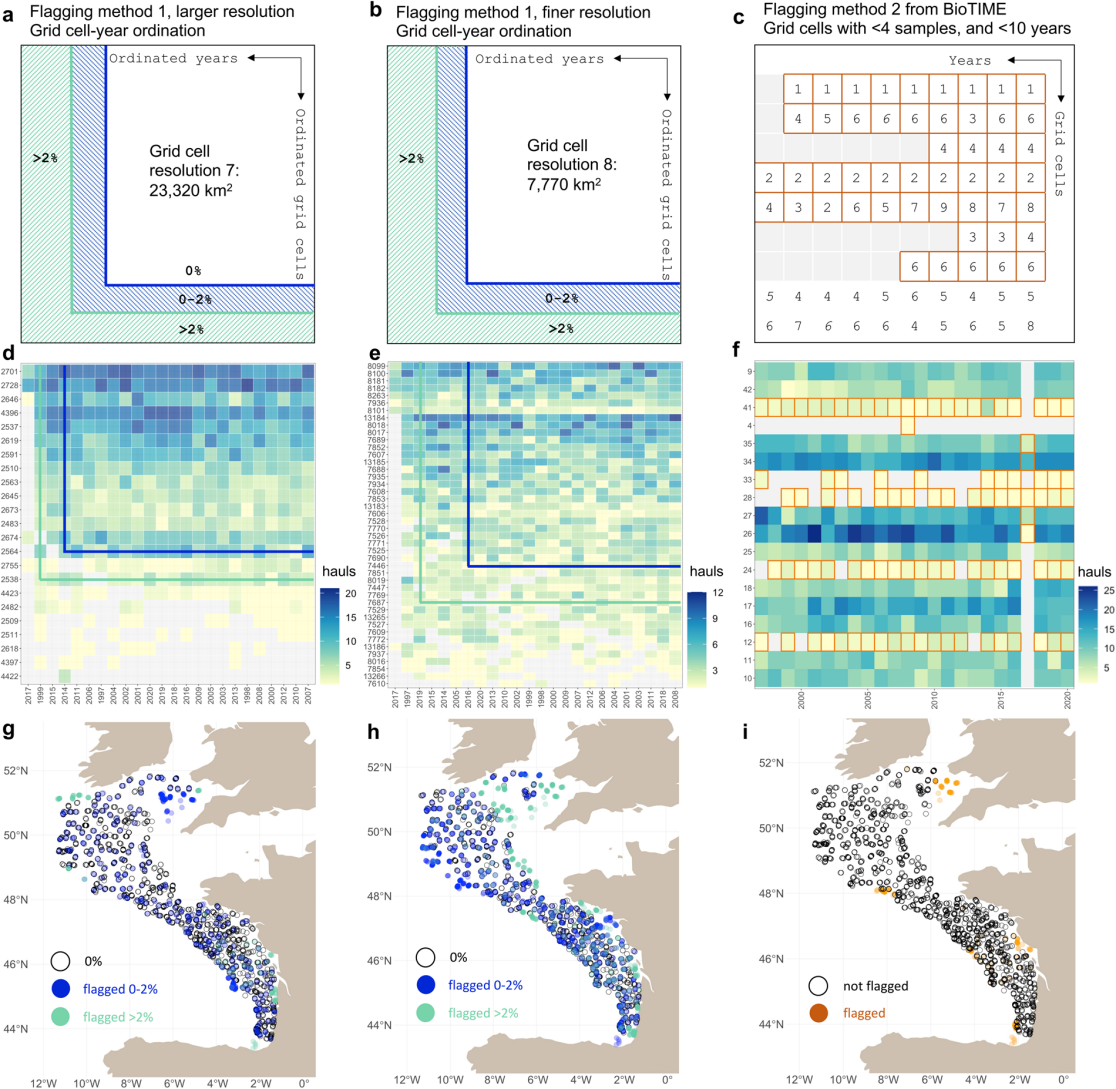
Spatio-temporal flagging methods illustrated with the EVHOE survey sampling the Celtic Sea and Bay of Biscay continental shelf and slope. Conceptual diagrams illustrate the general method applied to each survey unit (i.e., a combination of survey and seasons and/or quarter) for the first flagging method at coarser (**a**) and finer (**b**) spatial resolutions and for the second flagging method from BioTIME (**c**). The ordination and flagging results are illustrated on the sampling effort time-series for the EVHOE SBTS for each method (**d**–**f**). The lines in the plots (**d,e**) separate the well-sampled grid cells and years (in the top-right corner) from the grid cells (below the horizontal line) and year (to the left of the vertical line) flagged for potential removal. Lines are plotted according to the 0% and 2% thresholds (blue and green, respectively). Maps display the location of flagged hauls for the EVHOE survey according to the two flagging methods, spatial resolutions, and thresholds when applicable (**g**–**i**).

**Fig. 4:**
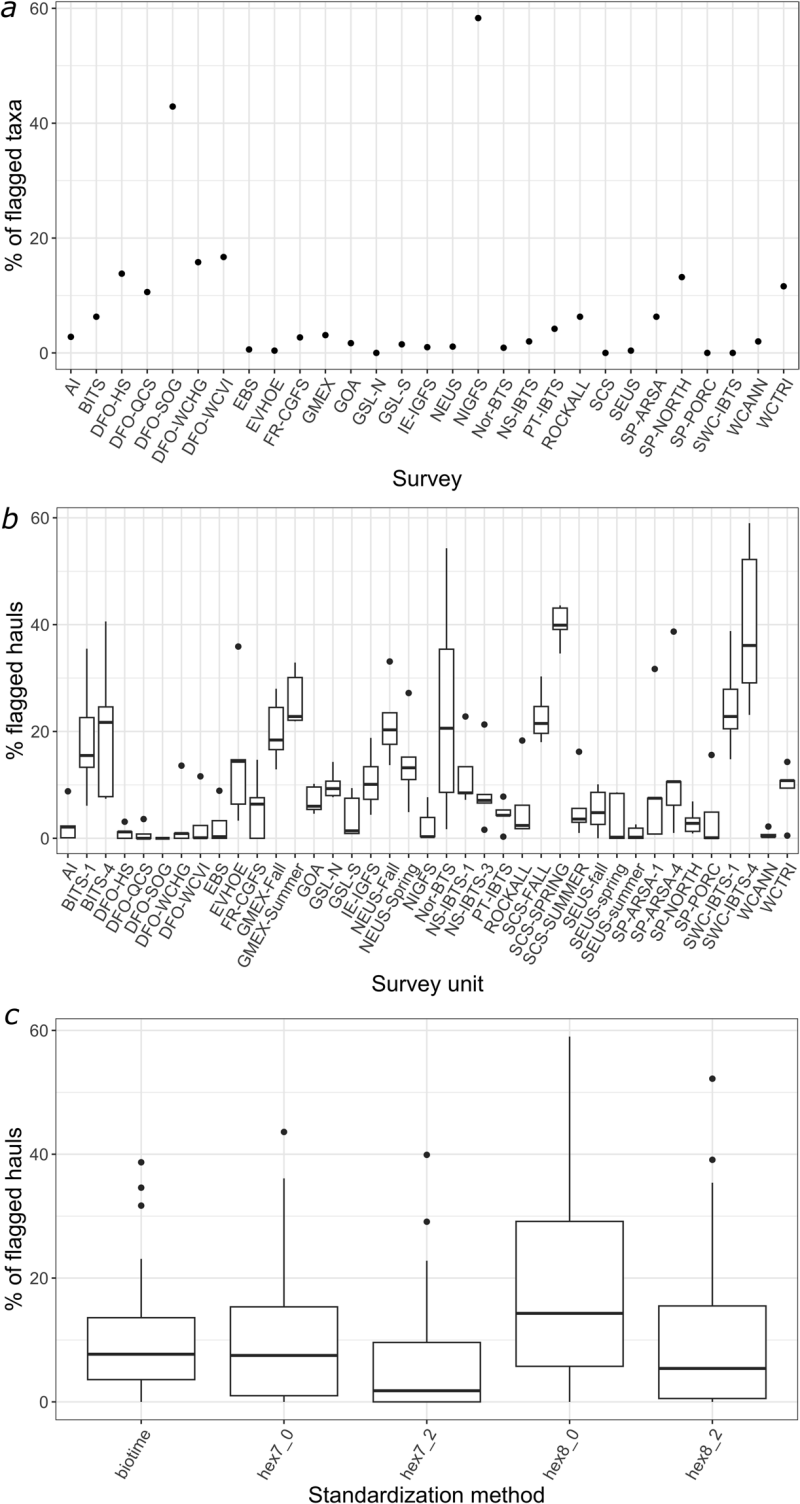
Summary of flagging results across surveys and methods. (**a**) Percentage of flagged taxa per survey (**b**) Percentage of flagged hauls per survey across spatio-temporal standardization methods (**c**) percentage of flagged hauls per method across surveys. Flagged hauls are suggested for removal to help create a stable spatial footprint through time.

Although SBTS provide high spatio-temporal data coverage, the spatial footprint of each survey often varies through time because of logistical constraints or opportunities in a particular year. We established a flagging system to identify a spatial footprint that has been consistently sampled through time. We followed two primary methods of ensuring spatio-temporally consistent coverage: (i) a spatial grid cell temporal coverage method; and (ii) a standardization method previously developed for the BioTime dataset^57–59^ (Fig. 3a–c). For (i), we gridded hauls separately on an equal-area hexagonal grid for each temporal survey unit (i.e., a temporal survey unit is a code defined by the combination of ‘survey’ and ‘season’ and/or ‘quarter’ from Supplementary Table S2, such as NS-IBTS quarter 1). We used the “dggridR” R package^60^ with spatial resolutions of 7 and 8, which correspond to hexagonal grid cells of approximately 23,320 km^2^ and 7,770 km^2^, respectively. Each combination of a grid cell and year it was sampled was termed a “grid cell-year”. We then identified the largest set of grid cells and years such that all retained grid cells were sampled in all retained years (a 0% missing threshold). We also identified the largest set of grid cells and years such that a maximum of 2% of all the grid cell-years of a temporal survey unit were missing (a 2% missing threshold). We flagged survey hauls not in the 0%/resolution 7 core set, not in the 0%/resolution 8 core set, not in the 2%/resolution 7 core set, or not in the 2%/resolution 8 core set (see conceptualized diagrams on Fig. 3a,b). Note that for these two maximization processes (0% and 2%), both grid cells and years could be flagged. For (ii), we gridded hauls based on a pre-designed grid for each temporal survey unit^59,61^. The resolution of the grid is specific to each survey and set as 1/5 of the latitudinal and longitudinal range. Each grid cell included haul locations (combination of latitude and longitude). First, a haul was flagged if its corresponding grid cell was not sampled at least 4 times in an individual year^61^. The minimum of 4 sampling events in a grid cell ensure that a grid cell is not biased by sampling noise of only a few sampling events. Second, grid cells (including at least 4 sampling locations per year) were removed if they included less than 10 years of sampling (Fig. 3c). 10 years of sampling are considered as the shortest time-series necessary for being able to perform temporal ecological analyses in this method^59,62^, and more generally in ecology^63^. In total, we associated each survey haul with five different sampling flags: 4 for method (i) and 1 for method (ii). End-users may wish to use one of these systems to standardize the spatial footprint of the surveys. The different methods remove somewhat different hauls and result in core datasets that are spatially consistent through time to different degrees (Fig. 4).

## Data Records

The final FISHGLOB_data included 216,548 unique sampled hauls across 29 surveys, recording presence, abundance and/or weight of 2,170 unique fish taxa. The dataset is available for download from the GitHub repository (https://github.com/AquaAuma/FishGlob_data/tree/main/outputs/Compiled_data) and a release available for download on Zenodo (https://zenodo.org/records/10218308)^64^. All surveys were identified with a unique code (‘survey’, Supplementary Table S2), with the full list detailed in Supplementary Table S1. All relevant focal variables related to the geolocation (‘latitude’, ‘longitude’, ‘stratum’, ‘station’, ‘stat_rec’, ‘continent’) and date of sampling (‘year’, ‘month’, ‘day’, ‘season’, ‘quarter’) are reported when relevant (Supplementary Table S2). For each survey, when available, we reported the abundances and weights per taxa from the raw data and standardized by the haul duration and swept area (Supplementary Table S2). The dataset included fields related to the taxonomy extracted from WoRMS (‘aphia_id’, ‘accepted_name’ and related classification), FishBase (‘SpecCode’), and the original reported names and codes from the raw datasets (‘verbatim_name’ and ‘verbatim_aphia_id’, respectively).

The spatial footprint of the surveys spanned almost continually across three continental shelves of the northern hemisphere, from the Pacific Northwest Coast to the Northeast Atlantic and the Northwest Atlantic (Fig. 2). SBTS ranged from relatively long time-series (e.g., the North Sea NS-IBTS and Northeast US NEUS began in the 1960s), to short time-series (e.g., the Strait of Georgia DFO-SOG began in 2012 and Irish Sea NIGFS began in 2005, Supplementary Table S1, Fig. 2). Most surveys sampled their regions annually, but some surveys are conducted every two (e.g., DFO-SOG, DFO-WCVI, DFO-QCS, DFO-HS, DFO-WCHG) or three years (e.g., WCTRI). Surveys also varied in their intra-annual sampling; the ‘survey_unit’ column field (Supplementary Table S2) indicates which surveys operated in multiple seasons (e.g., BITS, NS-IBTS, SWC-IBTS, NEUS, SEUS, SCS, SP-ARSA). In terms of spatial extent and length of time-series, the most extensive surveys were the Northeast US (NEUS), the North Sea (NS-IBTS), the Norwegian Barents Sea (Nor-BTS), the Gulf of Alaska (GOA), and the Gulf of St Lawrence North and South (GSL-S, GSL-N).

The integrated datasets also include taxonomic and spatio-temporal flags, to help users in conducting spatio-temporal studies with consistent taxa, as well as consistent regional spatial footprints (see fields at the end of Supplementary Table S2). On average, 7.8% of taxa per survey were flagged as potentially of concern across surveys (Fig. 4a). Some surveys showed a rather large proportion of taxa flagged, especially for shorter time-series. This was the case of the Irish Sea survey (NIGFS), for which has 16 years of sampling, and for which 58% of taxa were flagged (56 out of 96 sampled taxa). However, several surveys showed less than 1% of taxa flagged, as was the case for the Scotian Shelf, the Northern Gulf of Saint Lawrence, the Eastern Bering Sea, the Porcupine bank, the Scottish West Coast, the Barents Sea, the Southeast US, and the Celtic Sea and Bay of Biscay survey regions (SCS, GSL-N, EBS, SP-PORC, SWC-IBTS, Nor-BTS, SEUS, and EVHOE, respectively).

We detailed the results of the spatio-temporal flags using two different methods and spatial scales for the Celtic Sea and Bay of Biscay (EVHOE) as an example of options for trimming out hauls to ensure a consistent spatial survey footprint over time (Fig. 3). When a standardization method was applied to that survey, some hauls were flagged because some grid cells and years were not consistently sampled over time or space. With the first trimming method, 14.7% of all the hauls done in EVHOE survey were flagged under a 0% missing data threshold, and 3.3% under a 2% threshold at the coarser grid cell resolution (Fig. 3d,g). At the finer spatial resolution, the percentage of flagged hauls was higher: 35.9% under the 0% threshold, and 14.4% under the 2% threshold (Fig. 3e,h). The second trimming method developed for the BioTIME dataset led to 6.4% of hauls flagged for removal (Fig. 3i).

The percentage of hauls flagged for spatio-temporal coverage that was not constant across surveys and methods and may influence biodiversity studies using metrics sensitive to geolocation and temporal survey fluctuations (Fig. 4b,c). The winter Scottish West Coast survey (SWC-IBTS quarter 4) had the largest proportion of flagged hauls, indicating its spatio-temporal survey footprint is not very constant (from 23.1% to 59% of hauls were flagged, depending on the method). On the contrary, the Eastern Bering Sea (EBS) has been sampled following a strict survey design with a remarkable regularity, and the standardization methods led to very few flagged hauls (from 0% to 8.9% of hauls were flagged across methods). The most liberal approach was trimming with the coarser spatial grid cell resolution (7) and allowing 2% missing grid cell-years (6.6% of flagged hauls on average across survey units, Fig. 4c), while the most restrictive was the finer grid cell resolution (8) and the 0% threshold (17.9% of flagged hauls on average across survey units).

## Technical Validation

### General QAQC steps

Quality checks common to all SBTS included checking taxonomic consistency homogenization steps to make sure taxonomic names were valid and most accurate. QAQC included consulting manuals and experts to incorporate survey-specific knowledge in the dataset. This incorporation was done on an iterative basis as issues were identified, such as when investigating the taxonomic flags. The taxonomic flags have been a useful method to identify taxonomic splits and lumps over time, as well as other inconsistencies in how species are identified in the field. We only included hauls with consistent gears applied within each survey region and the best-sampled season(s) or months of the year (Supplementary Table S1). Other standard checks included checks that abundances and weights were either null, positive, or recorded as missing, but not negative. Outliers and value ranges were not constrained is the dataset to maximize usage, as users may be interested in a part of the dataset and apply their own filters. However, value ranges and outliers can be already detected in the survey summaries described below.

### Survey-specific QAQC steps

We performed several survey-specific quality assurance and quality check steps for ICES-sourced SBTS. Important technical validation aspects included:

- comparing our swept area estimation with the one developed by the ICES^65^ (Supplementary Figure 1). Some differences were observed, especially for the Baltic Sea (BITS survey) for which uncertainties were noted. However, swept area values remained in the same range overall from both sources, validating our methods.
- comparing the abundances and weights estimated from the abundance-at-length calculation with the abundances and weights reported at the taxa-haul level. We found very strong correlations for the abundances (all close to 1), while weights were sometimes different. These differences may be due to unresolved inconsistencies in the datasets, such as misreported units for weights or length measurements. This issue has already been flagged by ICES for historical data from the Baltic and North Seas (BITS and NS-IBTS, respectively), and is an ongoing issue for these datasets. For this reason, we preferred to include the recalculated weights from abundance-at-length from ICES datasets.

### Survey summaries

For each bottom-trawl survey, we generated summaries using RMarkdown^66^ to display sampling characteristics, distributions of variables, and standardization of results. These summary files were used to visually perform quality control on the data generated per survey and were carefully checked before finalizing the dataset. This quality control step complemented the quality filters step at the beginning (see Data standardization section), and these summaries should be examined by end-users to help assess data fitness for their needs. Each summary contains important survey information (data provider, temporal extent, survey region) and the cleaning R code for transparency of metadata and survey processing. Then, an overview of the survey information is provided that includes:

1. snippet of the survey data table
2. the number of hauls per year to verify/be aware of the consistency in sampling from the start to the end survey year
3. the distribution of the sampling variables per year (e.g., swept area, haul duration, sampling depth) to verify/be aware of the consistency in the range of the sampling variable from the start to the end survey year
4. the biological variables per year (e.g., abundance and weight and related standardized units CPUA and CPUE)
5. the distribution of abundance and weight values per year to identify/be aware of potential outliers in the dataset from the start to the end survey year
6. the relationship between the biological variables and the swept area to verify/be aware of the effect of the swept area on the abundance and/or biomass from the start to the end survey year
7. the abundance or weight trends of the six most abundant taxa to verify/be aware of the abundance or weight trend. These trends were verified against known trends from available stock assessments or other available platforms making species-specific temporal trends available for the same survey regions and datasets (https://james-thorson.shinyapps.io/FishViz/)
8. the map of the survey haul locations to verify/be aware of the spatial distribution in the sampling events for all survey years
9. the taxonomic flagging method results to verify/be aware of the taxa that are identified as not consistently sampled from the start to the end survey year
10. the spatio-temporal flagging method results to verify/be aware of the consistency in spatio-temporal data coverage

An overall summary of FISHGLOB_data was generated to verify the distribution of some variables across surveys. In particular, this summary shows the distribution of the sampling and biological variables per year. This visualization helped to check whether variables were relatively constant throughout the survey period. For instance, one would not expect a large change in the swept area, sampling depth, or haul duration over time. All summaries are available at: https://github.com/AquaAuma/FishGlob_data/tree/main/summary and provide overviews of each individual survey. We used these summaries early on to detect and correct the quality control issues in the processed dataset, and we encourage users to consult these summaries before using the data in their own analyses.

## Usage Notes

The FISHGLOB_data spatio-temporal fish community dataset was designed to maximize utility for a variety of purposes. However, misunderstanding or misuse of this dataset can easily produce meaningless results. Users should be fully aware of the dataset limitations. We advise paying attention to the following categories and points:

### Choice of focal variables

- abundance or weight variables: not all surveys include abundance and weight data. Choosing one or the other will automatically remove several surveys. Users should think carefully about which measure is most relevant to their questions. Biodiversity community metrics, for example, can produce substantially different results if calculated with abundance vs biomass^67^.
- standardization of focal variables: users should be aware the methods for computing the swept areas for the ICES DATRAS SBTS represent one way of calculating the swept area^54,56^, and others have been made available since the development of this dataset (https://www.ices.dk).
- taxa presence-absence: each survey dataset can be transformed to a taxa “presence-absence” dataset, meaning that each taxon that occurs in the survey but is not found in a sampling haul (but found elsewhere in the same survey) may be considered as an absence. This assumption is only valid for taxa that a survey consistently identified in all years. In other words, a taxon only recently described cannot be considered as absence in earlier years.

### Additional survey variable filters

- survey and seasons: some surveys include sampling at different seasons (reflected by the ‘month’, ‘season’, ‘quarter’ variables, Supplementary Table S2), and users should decide whether to consider and include all seasons for all surveys. Surveys do not necessarily sample the same seasons in all years, such that the phenology of the survey may change across the years. For some analyses, such phenological changes may be problematic. Also, some surveys are conducted in the winter season, meaning that sampling occurs at end of year X and the beginning of year X + 1. In such cases, the ‘year’ variable is not the best way to differentiate survey years.
- other time variables: the sampling time of the day might affect the species assemblages reported in the datasets. Users should consider whether this would be important in their application of the dataset, notably concerning the diel vertical migration and the different assemblages between day and night. This aspect, however, has not been closely investigated in the dataset, and the information of daytime/nighttime was not always available depending on the surveys.
- consistent spatial area: if it is important that a consistent spatial area has been surveyed through time, we advise filtering the haul data based on one of the spatial standardization methods included in the dataset (see Optional Standardization flagging section). Variation in spatial footprint can drive variation in observed community composition, species distribution, species abundance, and other biological measures calculated from SBTS. However, we also advise users to check with survey experts from individual regions to contextualize further the change in spatial footprint over time depending on the use case, to make sure the filters are appropriately interpreted. Regional expert contacts can be found in Maureaud *et al*.^13^.
- temporal extents across surveys: surveys do not cover identical time-series, and users should explore the temporal extent of each survey and decide which years and surveys to use for their research questions. For instance, years could be constrained to the most recent time-series (2006–2020) to include all surveys^26,28^. In addition, some years were not sampled within a survey, while other surveys were conducted at variable intervals over time. For instance, DFO-WCVI is only conducted every two years, while NS-IBTS is conducted twice a year (Supplementary Table S1, Fig. 2).
- sampling characteristics: all sampling variables may be further constrained to certain ranges and filtered. For instance, it is common to exclude sampling depths shallower than 20 meters to remove the very nearshore communities from the surveys^19^.

### Additional taxonomic filters and disclaimers

- pelagic taxa: SBTS catch both pelagic and demersal fish taxa, and researchers should consider whether keeping pelagic taxa makes sense for their questions. Pelagic taxa can be identified by linking FISHGLOB_data with FishBase^49^ using the ‘SpecCode’ (Supplementary Table S2). The catchability of pelagic fish is much lower in the bottom-trawl surveys^54^. Alternatively, researchers may be able to apply catchability correction factors per water column habitat for more consistent abundance or weight estimation within surveys^27^.
- inconsistent/incomplete taxonomic reporting: some surveys do not appear to report most fish taxa consistently over time, such as PT-IBTS (low number of taxa on average per year), SP-ARSA (with flag on year 2018), SP-NORTH (1990–1992), SP-PORC (year 2014)
- taxa rank: the fish taxa included in the dataset includes higher ranks than the species level (e.g., family, genus), and users should decide whether all ranks should be kept or restricted to certain ranks.
- flagged taxa: these could be filtered if users think they should not be included in the dataset. Consulting with survey contacts^13,46^ may be useful here. We encourage opening new issues in the GitHub repository (https://github.com/AquaAuma/FishGlob_data)^64^ to document new information on taxa to include, exclude, or flag. Depending on sources, taxonomic treatments recommended per survey are not the same and users need to carefully check the taxonomic treatment adapted for their own use^23,68,69^.
- accepted name: sometimes the ‘verbatim_name’ or ‘verbatim_aphia_id’ reported in a survey haul is not identical to the ‘accepted_name’ due to changes in taxonomic treatments, or to knowledge in surveys of misidentifications. This may sometimes create duplicate taxa in survey hauls. For instance, in the North Sea SBTS data, we assigned the genus *Argentina* instead of the reported *Argentina Sphyraena* or *Argentina silus* because species distinction has been considered too difficult to be certain of the identification. However, reported ‘verbatim_aphia_id’ still includes the original reported taxonomic information.

### Scale of analysis

- spatial scale: users can decide to work at different scales, such as the exact survey locations at the haul level, the aggregation within a grid (two spatial resolutions are provided in the dataset), or the aggregation at the survey region level indicated with the ‘survey’ column.
- comparison among surveys: because of differences in the precise gear and sampling design in different surveys, and their ability to catch different species (e.g., catchability) differs. Abundance and weights are not directly comparable across surveys, even after per unit effort and per unit area corrections have been applied.

### Survey-specific warnings

A list of survey-specific aspects has been created to warn and advise users in working with these surveys. These notes are mostly useful in the integration process and may help users building a better understanding of the data source for their own usage and are available online at: https://github.com/AquaAuma/FishGlob_data/tree/main/metadata_docs.

### Overall guidance

The dataset presented here is only one way of treating the SBTS data that hopefully maximizes diverse usages, but it remains the responsibility of the user to make sure to use the dataset properly. We kept the dataset unfiltered, meaning that it maximizes potential applications, but does not correct for inconsistencies in the datasets themselves. Finally, we encourage users and experts to provide feedback on survey data treatment to further improve this cross-continent dataset and integrated methodology, either by getting directly in touch with the authors, or by submitting GitHub issues. A user disclaimer also summarizes guidelines in using these data on GitHub.

We highly encourage users to cite this paper along with primary sources corresponding to the SBTS included studies to retain credit to primary institutions and experts publishing the raw versions of the datasets (all datasets are cited in Supplementary Table S1) and cite this paper for the integration methods developed to gather regional surveys together.

### Supplementary information

Supplementary Information

## Data Availability

All the code to generate FISHGLOB_data from the raw datasets can be accessed here: https://github.com/AquaAuma/FishGlob_data and archived on Zenodo for download https://zenodo.org/records/10218308.
