# Supplementary material for: FISHGLOB_data: an integrated dataset of fish biodiversity sampled with scientific bottom-trawl surveys
Source: Sci Data. 2024 Jan 4;11:24. doi: 10.1038/s41597-023-02866-w (PMC10766603; doi:10.1038/s41597-023-02866-w)
Supplement: Supplementary file 1 — Supplementary Information [file 41597_2023_2866_MOESM1_ESM.pdf]

Supplementary Information

Table of contents

Figure S1: Comparison of swept area estimates.
Table S1: Survey data sources and metadata summaries.
Table S2: Description of fields included in the integrated dataset.
Table S3: Survey data sources describing the method to obtain haul duration, swept area, abundance, and weight for each SBTS.

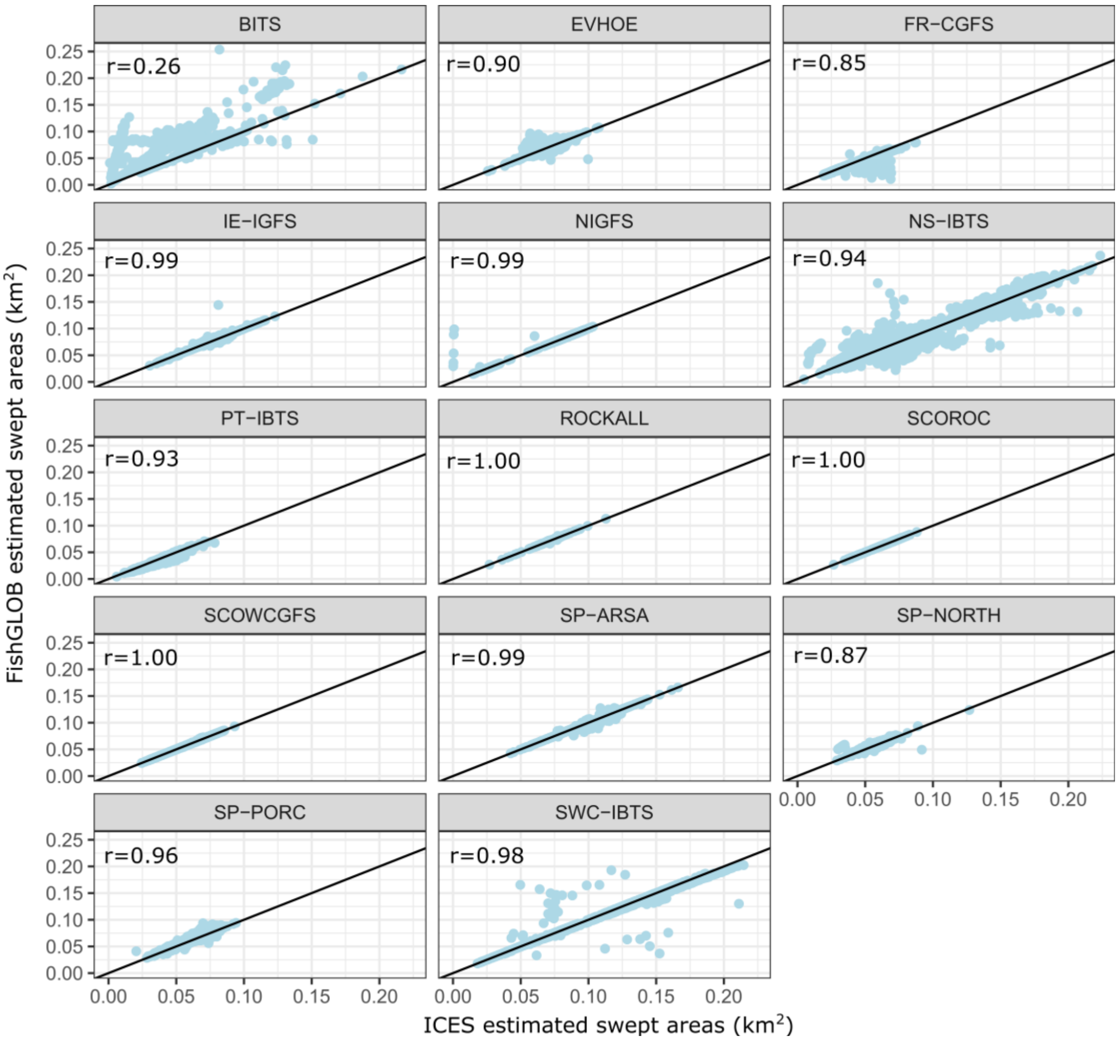

Figure S1: Comparison of swept area estimates in km^2 in the FISHGLOB_data (y-axis) and the ICES DATRAS (x-axis) [1]. Each inset graph is an ICES DATRAS SBTS listed in Table 1, where the Pearson's correlation is indicated in the top left corner.

Table S1: Survey data sources and metadata summaries. "GOV" stands for "Grande Ouverture Verticale" (i.e., large vertical opening), "NCT" stands for "Norwegian Campbell Trawl 1800/96" "ROT" stands for "Rock hopper otter trawl", TVL stands for "Small TV-3", TVS stands for "Large TV-3", "BAK" stands for "Baka trawl 44/60" and "PROB" stands for "Porcupine baca 40/52". The source and reference column indicates documents with more information about the sampling gear and survey designs. Months are numbered from January (1) to December (12).

survey	area	temporal extent	number of years	months	number of hauls	number of fish taxa	gear type	source and reference
AI	Aleutian Islands	1983-2018	14	5, 6, 7, 8, 9, 10, 11	5473	251	Poly Nor'Eastern trawl[a]	NOAA[2,3]
BITS	Baltic Sea	1992-2020	29	2, 3, 9, 10, 11, 12	10423	126	multiple gears (GOV, TVL, TVS)[b]	ICES[4,5]
DFO-HS	Canada, Hecate Strait	2005-2019	8	5, 6	1266	167	Atlantic Western IIa box trawl with rockhopper foot gear[c]	DFO[6,7]
DFO-QCS	Canada, Queen Charlotte	2003-2019	10	7, 8	2384	199	Atlantic Western IIa box trawl with rockhopper foot gear[d]	DFO[6,8]
DFO-SOG	Strait of Georgia	2012-2015	2	3, 5	93	84	Yankee 36 Trawl net connected to 1,135 kg U.S.A. Jet doors and equipped with a hard-bottom footrope[e]	DFO[6,9]
DFO-WCHG	Canada, West Coast Haida Gwaii	2006-2018	8	8, 9, 10	882	177	Atlantic Western IIA box trawl net connected to 963 kg Thyboron Type II heavy duty 107 doors with rockhopper footgear[f]	DFO[6,10]

DFO-WCVI	Canada, West Coast Vancouver Island	2004-2018	8	5, 6	1176	180	Atlantic Western IIA box trawl net connected to 963 kg Thyboron Type II heavy duty 107 doors with rockhopper footgear[g]	DFO[6,11]
EBS	Eastern Bering Sea	1982-2019	38	5, 6, 7, 8, 9	14089	170	38-112	NOAA[3,12]
EVHOE	Bay of Biscay	1997-2020	24	10, 11, 12	3283	229	GOV 36/47	ICES[5,13]
FR-CGFS	Eastern English Channel	1998-2020	23	9, 10, 11	2071	113	GOV 19/25 (before 2014), GOV 36/47 (2015-present)	ICES[5,13]
GMEX	Gulf of Mexico	1987-2020	34	5, 6, 7, 8, 9, 10, 11, 12	19410	809	two-seam bottom-trawl (27.4 m length footrope), fished with W-style trawl doors (682 kg each, 3.5 m^2)[h]	SEAMAP[14,15]
GOA	Gulf of Alaska	1984-2019	16	5, 6, 7, 8, 9, 10	11602	360	Poly Nor'eastern trawl[i]	NOAA[3]
GSL-N	Gulf of Saint Lawrence North	1980-2019	39	1, 2, 4, 6, 7, 8, 9, 10, 11, 12	17669	162	multiple gears: Western IIA trawl (1984-1990), URI trawl (1990-2005), Campelen 1800 trawl (2004-present)[j]	DFO[16–18]
GSL-S	Gulf of Saint Lawrence South	1970-2019	49	8, 9, 10	6879	134	multiple gears: Yankee-36 trawl (1971-1985), Western IIA trawl (1985-1991), Western IIA trawl (1992-present)[k]	DFO[19]

IE-IGFS	Ireland Shelf Sea	2003-2020	18	9, 10, 11, 12	2880	195	GOV 36/47	ICES[5,13]
NEUS	Northeast US	1963-2020	58	2, 3, 4, 5, 6, 9, 10, 11, 12	36792	561	Multiple gears: #36 Yankee trawl, #41 Yankee, ¾ Yankee with conversion factors[20]	NOAA[14,21]
NIGFS	Irish Sea	2005-2020	16	2, 3, 4, 10, 11	1487	96	ROT	ICES[5,13]
NorBTS	Norwegian survey in the Barents Sea	2004--2021	18	8, 9, 10	3868	108	Campelen 1800 shrimp trawl with 20-, 22-, or 35-mm mesh size	IMR[22–24]
NS-IBTS	North Sea	1967-2020	53	1, 2, 3, 6, 7, 8, 9	25217	245	Multiple gears before 1983, GOV 36/47 standard since 1983 in Q1, and since 1998 by all vessels in Q3. GOV filtered in the final dataset.	ICES[5,25]
PT-IBTS	Portugal Shelf Sea	2002-2018	14	9, 10, 11	1179	191	NCT	ICES[5,13]
ROCKALL	Rockall Plateau	1999-2020	19	8, 9	779	79	GOV 36/47	ICES[5,13]
SEUS	Southeast US	1989-2019	31	4, 5, 6, 7, 8, 9, 10, 11	8520	251	42' semi-balloon trawl with 8'x40" chain doors towed[l]	SEAMAP[14,15]
SP-ARSA	Gulf of Cadiz	2002-2020	19	2, 3, 10, 11, 12	1301	159	BAK	ICES[5,13]
SP-NORTH	North of Spain	1990-2019	29	8, 9, 10, 11	2762	197	BAK	ICES[5,13]
SP-PORC	Porcupine Bank	2001-2019	19	8, 9, 10	1539	186	PORB	ICES[5,13]

SCS	Scotian Shelf	1970-2020	51	2, 3, 4, 6, 7, 8, 9, 10, 11, 12	14606	377	Western IIA trawl, US 4 seam 3 bridle survey trawl, Yankee #36 otter trawl	DFO[14,26]
SWC-IBTS	Scotland Shelf Sea	1985-2020	36	1, 2, 3, 10, 11, 12	3850	154	GOV 36/47	ICES[5,13]
WCANN	West Coast US	2003-2018	16	5, 6, 7, 8, 9, 10	10518	458	Four-panel Aberdeen 85/104' bottom-trawl equipped with net mensuration gear[m]	NOAA[14,27]
WCTRI	Triennial West Coast US	1977-2004	10	1, 2, 5	4551	303	Poly Nor'Eastern trawl with a footrope equipped with roller bobbins to allow fishing in rough habitat[m]	NOAA[14]

[a] https://www.psmfc.org/tsc2/Presentations/AFSC_Aleutian_Gulf_Shelf.pdf
[b] https://datras.ices.dk/Documents/Manuals/Addendum_1_WGBIFS_BITS_Manual_2011.pdf
[c] https://waves-vagues.dfo-mpo.gc.ca/library-bibliotheque/40769835.pdf
[d] https://publications.gc.ca/collections/collection_2019/mpo-dfo/Fs97-4-3150-eng.pdf
[e] https://publications.gc.ca/collections/collection_2014/mpo-dfo/Fs97-6-3056-eng.pdf
[f] https://waves-vagues.dfo-mpo.gc.ca/library-bibliotheque/40891586.pdf
[g] https://publications.gc.ca/collections/collection_2020/mpo-dfo/Fs97-4-3195-eng.pdf
[h] https://aquadocs.org/bitstream/handle/1834/26277/mfr7242.pdf?sequence=1&isAllowed=y
[I] https://meetings.npfmc.org/CommentReview/DownloadFile?p=afc47092-2c24-4d81-81a6-5af746dd46ee.pdf&fileName=PRESENTATION_GOA2019SurveyPlanTeamPalsson.pdf
[j] https://publications.gc.ca/collections/collection_2022/mpo-dfo/Fs97-6-3454-eng.pdf
[k] https://publications.gc.ca/collections/collection_2012/mpo-dfo/Fs97-6-2505-eng.pdf
[l] https://sedarweb.org/documents/s27rd05-fishery-independent-sampling-seamap-trawl/
[m] https://www.psmfc.org/tsc2/Presentations/NWFSC_West_Coast.pdf

Table S2: Description of fields included in the integrated dataset.

field	unit	type	description
survey		character	survey codes from Table 1
source		character	IMR, NOAA, ICES, DFO
timestamp		numeric	year and month the data were downloaded from the original sources
haul_id		character	unique ID created as Survey/Year/Quarter/Country/Ship/Gear/StNo/HaulNo for ICES surveys or any other unique combination including long/lat
country		character	country names
sub_area		character	Sub-area within survey (self-reported by data provider if the sampling design uses sub-areas; NA if not available)
continent		character	europe, n_america, s_america, asia, africa, oceania, arctic, or antarctica
stat_rec		character	ICES statistical rectangles[28] (only available for DATRAS surveys)
station		character	Sampling station (self-reported by data provider if the sampling design uses stations; NA if not available)
stratum		character	Unique identifier for the sampling stratum (self-reported by data provider if the sampling design uses strata; NA if not available). A stratum is a sub-area within a survey that is consistently sampled, i.e., in a stratified random sampling design.
year		integer	year of sampling
month		integer	month of sampling, from 1 to 12
day		integer	day of sampling
quarter		integer	1 to 4, 1=January to March, 2=April to June, etc. used for European surveys, following DATRAS codes[29]
season		character	Winter, Spring, Summer, Autumn used for North American surveys

latitude	degrees, geographic coordinate system WGS84	numeric	haul latitude, ideally at beginning of the haul
longitude	degrees, geographic coordinate system WGS84	numeric	haul longitude, ideally at beginning of the haul
haul_dur	hours	numeric	haul duration
area_swept	km^2	numeric	haul swept area, NA if not available
gear		character	name of gear used for sampling, NA if not available
depth	meters	numeric	sampling depth, ideally at beginning of the haul
sbt	degrees C	numeric	sampling sea bottom temperature reported by the survey, NA if not available
sst	degrees C	numeric	sampling sea surface temperature reported by the survey, NA if not available
num	number of individuals	numeric	number of individuals sampled
num_cpue	number of individuals/hour	numeric	num/haul_dur
num_cpua	number of individuals/km^2	numeric	num/area_swept
wgt	kg	numeric	weight of sampled individuals
wgt_cpue	kg/hour	numeric	wgt/haul_dur
wgt_cpua	kg/km^2	numeric	wgt/area_swept
verbatim_name		character	Taxon name from the data received, NA if not available
verbatim_aphia_id		character	AphiaID (from WoRMS) from the data received, NA if not available
accepted_name		character	accepted scientific name of the taxa (verified with WoRMS[30])
aphia_id		character	WoRMS AphiaID valid name code[30]

SpecCode		numeric	FishBase species code[31,32]
kingdom		character	kingdom retrieved from WoRMS[30]
phylum		character	phylum retrieved from WoRMS[30]
class		character	class retrieved from WoRMS[30]
order		character	order retrieved from WoRMS[30]
family		character	family retrieved from WoRMS[30]
genus		character	genus retrieved from WoRMS[30]
rank		character	rank retrieved from WoRMS[30]
survey_unit		character	combination of survey with quarter or season (useful for BITS, NS-IBTS, SWC-IBTS, NEUS, SEUS, SCS)
trimming_hex7_0		character	TRUE if haul is flagged, for potential removal under hexagonal grid cell size 7 built with the dggridR package[33], 0% threshold, NA if not
trimming_hex7_2		character	TRUE if haul is flagged, for potential removal under the hexagonal grid size 7 built with the dggridR package[33], 2% threshold, NA if not
trimming_hex8_0		character	TRUE if haul is flagged, for potential removal under the hexagonal grid size 8 built with the dggridR package[33], 0% threshold, NA if not
trimming_hex8_2		character	TRUE if haul is flagged, for potential removal under the hexagonal grid size 8 built with the dggridR package[33], 2% threshold, NA if not
trimming_biotime		character	TRUE if haul is flagged for potential removal under the BioTIME method[34,35], NA if not
flag_taxa		character	TRUE if taxon is flagged under the OceanAdapt method[36], NA if not

Table S3: Survey data sources describing the method to obtain haul duration, swept area, abundance, and weight for each SBTS.

survey	area	haul duration	swept area	abundance	weight
AI	Aleutian Islands	Data obtained in effort format directly from source, no haul duration in FISHGLOB_data	Data obtained in effort format directly from source, no swept area in FISHGLOB_data	Reported in effort format in survey data	Reported in effort format in survey data
BITS	Baltic Sea	Measured and reported in the survey data	Calculated from reported haul-specific variables or predicted from linear models[a]	Counted and reported in survey data	Estimated from abundance at length data reported in survey data and length-weight relationships from FishBase[b]
DFO-HS	Canada, Hecate Strait	Measured and reported in the survey data	Calculated from reported trawl door spread and distance towed	Counted and reported in survey data	Measured and reported in survey data
DFO-QCS	Canada, Queen Charlotte	Measured and reported in the survey data	Calculated from reported trawl door spread and distance towed	Counted and reported in survey data	Measured and reported in survey data
DFO-SOG	Strait of Georgia	Measured and reported in the survey data	Calculated from reported trawl door spread and distance towed	Counted and reported in survey data	Measured and reported in survey data
DFO-WCHG	Canada, West Coast Haida Gwaii	Measured and reported in the survey data	Calculated from reported trawl door spread and distance towed	Counted and reported in survey data	Measured and reported in survey data

DFO-WCVI	Canada, West Coast Vancouver Island	Measured and reported in the survey data	Calculated from reported trawl door spread and distance towed	Counted and reported in survey data	Measured and reported in survey data
EBS	Eastern Bering Sea	Data obtained in effort format directly from source, no haul duration in FISHGLOB_data	Data obtained in effort format directly from source, no swept area in FISHGLOB_data	Reported in effort format in survey data.	Reported in effort format in survey data.
EVHOE	Bay of Biscay	Measured and reported in the survey data	Calculated from reported haul-specific variables or predicted from linear models[a]	Counted and reported in survey data	Estimated from abundance at length data reported in survey data and length-weight relationships from FishBase[b]
FR-CGFS	Eastern English Channel	Measured and reported in the survey data	Calculated from reported haul-specific variables or predicted from linear models[a]	Counted and reported in survey data	Estimated from abundance at length data reported in survey data and length-weight relationships from FishBase[b]
GMEX	Gulf of Mexico	Measured and reported in the survey data	Calculated from provided vessel speed, tow duration, and width of gear in survey data	Counted and reported in survey	Measured and reported in survey data
GOA	Gulf of Alaska	Data obtained in effort format directly from source, no haul duration in FISHGLOB_data	Data obtained in effort format directly from source, no swept area in FISHGLOB_data	Reported in effort format in survey data	Reported in effort format in survey data
GSL-N	Gulf of Saint Lawrence North	Measured and reported in the survey data	Calculated from provided tow distance and trawl width in survey data	Counted and reported in survey	Measured and reported in survey data

GSL-S	Gulf of Saint Lawrence South	All tows assumed to be 30 minutes (from data provider).	Gear specific swept area provided by Fisheries and Oceans Canada documentation[c]	Counted and reported in survey	Measured and reported in survey data
IE-IGFS	Ireland Shelf Sea	Measured and reported in the survey data	Calculated from reported haul-specific variables or predicted from linear models[a]	Counted and reported in survey data	Estimated from abundance at length data reported in survey data and length-weight relationships from FishBase[b]
NEUS	Northeast US	Measured and reported in survey data. FISHGLOB_data reflects calibrated values for gear and vessel change from 2008-2009, but the haul duration does not reflect this change. Therefore, for calculating effort, 30 minutes should be used as the denominator for all tows that users deem acceptable (near 30 minutes before 2009 and near 20 minutes from 2009 onward)	Average tow area for Albatross survey vessel (1963-2008) provided by Northeast Fisheries Science Center. FISHGLOB_data applies conversion to observations from Bigelow survey vessel from 2009 onwards, and therefore this same tow area value can be used for all abundance and weight measurements through the time series.	Counted and reported in survey data. Note that because of 2008-2009 vessel and gear change, effort is not calculated in FishGlob. FishGlob does apply conversion factors to observations from 2009 onward but recommends a close read of Miller et al. 2010[d] before using data and/or calculating effort.	Measured and reported in survey data. We recommend a close read of Miller et al. 2010[d] before using data and/or calculating effort.
NIGFS	Irish Sea	Measured and reported in the survey data	Calculated from reported haul-specific variables or predicted from linear models[a]	Counted and reported in survey data	Estimated from abundance at length data reported in survey data and length-weight relationships from FishBase[b]

NorBTS	Barents Sea	Measured and reported in the survey data	Calculated from the haul-specific trawled distance and an estimated constant door spread for the entire survey (50m)	Counted and reported in survey data	Measured and reported in survey data
NS-IBTS	North Sea	Measured and reported in the survey data	Calculated from reported haul-specific variables or predicted from linear models[a]	Counted and reported in survey data	Estimated from abundance at length data reported in survey data and length-weight relationships from FishBase[b]
PT-IBTS	Portugal Shelf Sea	Measured and reported in the survey data	Calculated from reported haul-specific variables or predicted from linear models[a]	Counted and reported in survey data	Estimated from abundance at length data reported in survey data and length-weight relationships from FishBase[b]
ROCKALL	Rockall Plateau	Measured and reported in the survey data	Calculated from reported haul-specific variables or predicted from linear models[a]	Counted and reported in survey data	Estimated from abundance at length data reported in survey data and length-weight relationships from FishBase[b]
SEUS	Southeast US	Measured and reported in the survey data	Calculated from provided start and end coordinates and net width	Counted and reported in survey data	Measured and reported in survey data
SP-ARSA	Gulf of Cadiz	Measured and reported in the survey data	Calculated from reported haul-specific variables or predicted from linear models[a]	Counted and reported in survey data	Estimated from abundance at length data reported in survey data and length-weight relationships from FishBase[b]

SP-NORTH	North of Spain	Measured and reported in the survey data	Calculated from reported haul-specific variables or predicted from linear models[a]	Counted and reported in survey data	Estimated from abundance at length data reported in survey data and length-weight relationships from FishBase[b]
SP-PORC	Porcupine Bank	Measured and reported in the survey data	Calculated from reported haul-specific variables or predicted from linear models[a]	Counted and reported in survey data	Estimated from abundance at length data reported in survey data and length-weight relationships from FishBase[b]
SCS	Scotian Shelf	Measured and reported in the survey data	Calculated from provided door spread and tow distance in survey data	Counted and reported in survey	Measured and reported in survey data
SWC-IBTS	Scotland Shelf Sea	Measured and reported in the survey data	Calculated from reported haul-specific variables or predicted from linear models[a]	Counted and reported in survey data	Estimated from abundance at length data reported in survey data and length-weight relationships from FishBase[b]
WCANN	West Coast US	Measured and reported in the survey data	Provided in survey data	Counted and reported in survey	Measured and reported in survey data
WCTRI	Triennial West Coast US	Measured and reported in the survey data	Calculated from provided net width and distance of tow	Counted and reported in survey data	Measured and reported in survey data

[a] https://github.com/AquaAuma/FishGlob_data/blob/main/cleaning_codes/source_DATRAS_wing_doorspread.R
[b] https://www.fishbase.se
[c] https://waves-vagues.dfo-mpo.gc.ca/Library/115732.pdf
[d] Miller, T. J., C. Das, P. J. Politis, A. S. Miller, S. M. Lucey, C. M. Legault, R. W. Brown, and P. J. Rago. 2010. "Estimation of Albatross IV to Henry B. Bigelow Calibration Factors." 10-05. Woods Hole, MA: National Marine Fisheries Service. https://www.nefsc.noaa.gov/publications/crd/crd1005/crd1005.pdf

Supplementary References

1. ICES. Data for the OSPAR request to generate swept area and abundance index outputs. (2021) doi:10.17895/ices.data.8286.
2. Von Szalay, P. G. (Paul G., Raring, N. W., Rooper, C. N. & Laman, E. A. Data report : 2016 Aleutian Islands bottom trawl survey. (2017) doi:10.7289/V5/TM-AFSC-349.
3. Fisheries, N. Alaska Groundfish Bottom Trawl Survey Data | NOAA Fisheries. *NOAA* https://www.fisheries.noaa.gov/alaska/commercial-fishing/alaska-groundfish-bottom-trawl-survey-data (2021).
4. ICES. *SISP 7 - Manual for the Baltic International Trawl Surveys (BITS)*. https://ices-library.figshare.com/articles/report/SISP_7_-_Manual_for_the_Baltic_International_Trawl_Surveys_BITS_/19050986/1 (2017) doi:10.17895/ices.pub.2883.
5. ICES. ICES Database on Trawl Surveys (DATRAS). (2023).
6. Anderson, S. C., Keppel, E. A. & Edwards, A. M. *A reproducible data synopsis for over 100 species of British Columbia groundfsh*. 321 p. (2019).
7. Secretariat, T. B. of C. & Secretariat, T. B. of C. Hecate Strait Synoptic Bottom Trawl Survey - Open Government Portal. https://open.canada.ca/data/en/dataset/780a1c02-1f9c-4994-bc70-a0e9ef8e3968.
8. Secretariat, T. B. of C. & Secretariat, T. B. of C. Queen Charlotte Sound Synoptic Bottom Trawl Survey - Open Government Portal. https://open.canada.ca/data/en/dataset/86af7918-c2ab-4f1a-ba83-94c9cebb0e6c.
9. Secretariat, T. B. of C. & Secretariat, T. B. of C. Strait of Georgia Synoptic Bottom Trawl Survey - Open Government Portal. https://open.canada.ca/data/en/dataset/d880ba18-8790-41a2-bf73-e9247380759b.
10. Secretariat, T. B. of C. & Secretariat, T. B. of C. West Coast Haida Gwaii Synoptic Bottom Trawl Survey - Open Government Portal. https://open.canada.ca/data/en/dataset/5ee30758-b1d6-49fe-8c4e-5136f4b39ad1.
11. Secretariat, T. B. of C. & Secretariat, T. B. of C. West Coast Vancouver Island Synoptic Bottom Trawl Survey - Open Government Portal. https://open.canada.ca/data/en/dataset/557e42ae-06fe-426d-8242-c3107670b1de.
12. Lauth, R. R., Dawson, E. J. & Conner, J. (Jason). Results of the 2017 eastern and northern Bering Sea continental shelf bottom trawl survey of groundfish and invertebrate fauna. doi:10.25923/H118-NW41.
13. ICES. *SISP 15 - Manual of the IBTS North Eastern Atlantic Surveys*. https://ices-library.figshare.com/articles/report/SISP_15_-_Manual_of_the_IBTS_North_Eastern_Atlantic_Surveys/19051037/1 (2017) doi:10.17895/ices.pub.3519.
14. Pinsky, M. L., Worm, B., Fogarty, M. J., Sarmiento, J. L. & Levin, S. A. Marine Taxa Track Local Climate Velocities. *Science* **341**, 1239–1242 (2013).
15. Gulf States Marine Fisheries Commission Southeast Area Monitoring and Assessment Program (SEAMAP) online access. https://seamap.gsmfc.org/.
16. Secretariat, T. B. of C. & Secretariat, T. B. of C. Evaluation of groundfish and shrimp annual multidisciplinary survey in the Estuary and northern Gulf of St. Lawrence (CCGS Alfred Needler 1990 - 2005) - Open Government Portal. https://open.canada.ca/data/en/dataset/4eaac443-24a8-4b37-9178-d7cce4eb7c7b.
17. Secretariat, T. B. of C. & Secretariat, T. B. of C. Evaluation of groundfish and shrimp annual multidisciplinary survey in the Estuary and northern Gulf of St. Lawrence (CCGS Teleost 2004 - 2021) - Open Government Portal. https://open.canada.ca/data/en/dataset/40381c35-4849-4f17-a8f3-707aa6a53a9d.
18. Secretariat, T. B. of C. & Secretariat, T. B. of C. Evaluation of groundfish annual multidisciplinary winter survey in the northern Gulf of St. Lawrence (MV Gadus

Atlantica 1978 - 1994) - Open Government Portal. https://open.canada.ca/data/en/dataset/4bbd03ce-ae48-4aaa-97ac-5594c2a3a6c2.
19. Secretariat, T. B. of C. & Secretariat, T. B. of C. NAFO Division 4T groundfish research vessel trawl survey (September Survey) dataset - Open Government Portal. https://open.canada.ca/data/en/dataset/1989de32-bc5d-c696-879c-54d422438e64.
20. Miller, T. J. *et al. Estimation of Albatross IV to Henry B. Bigelow calibration factors*. https://repository.library.noaa.gov/view/noaa/3726 (2010).
21. Azarovitz, T. R. A brief historical review of the Woods Hole Laboratory trawl survey time series. *Can. Spec. Publ. Fish. Aquat. Sci.* **58**, 62–67 (1981).
22. Johannesen, E. *et al. Fish diversity data from the Barents Sea Ecosystem Survey 2004-2019*. https://www.hi.no/en/hi/nettrapporter/rapport-fra-havforskningen-en-2021-15#sec-5 (2021).
23. Wienerroither, R. *et al. IMR/PINRO no 1 - 2011: Atlas of the Barents Sea Fishes*. https://www.hi.no/en/hi/nettrapporter/imrpinro/2011/atlas_august_2011_press_quality_til_web (2011).
24. Wienerroither, R., Langøy Mørk, H. & Johannesen, E. Barents Sea ecosystem survey fish diversity data export 2021 NO. (2023).
25. ICES. *SISP 10 – Manual for the North Sea International Bottom Trawl Surveys*. https://ices-library.figshare.com/articles/report/SISP_10_Manual_for_the_North_Sea_International_Bottom_Trawl_Surveys/19051361/1 (2020) doi:10.17895/ices.pub.7562.
26. Simon, J. E. & Comeau, P. *Summer distribution and abundance trends of species caught on the Scotian Shelf from 1970-92 by research vessel groundfish survey.* https://waves-vagues.dfo-mpo.gc.ca/library-bibliotheque/178892.pdf (1994).
27. Keller, A. A., Wallace, J. R. (John R. & Methot, R. D. The Northwest Fisheries Science Center's West Coast Groundfish Bottom Trawl Survey : history, design, and description. (2017) doi:10.7289/V5/TM-NWFSC-136.
28. ICES statistical rectangles. https://www.ices.dk/data/maps/Pages/ICES-statistical-rectangles.aspx.
29. ICES Reference Codes - RECO. https://vocab.ices.dk/?ref=1645.
30. WoRMS. WoRMS manual. https://www.marinespecies.org/aphia.php?p=manual (2022).
31. Froese, R. & Pauly, D. FishBase. World Wide Web electronic publication. https://www.fishbase.org/ (2022).
32. The SPECIES Table. https://www.fishbase.de/manual/english/fishbasethe_species_table.htm.
33. Barnes, R. & Sahr, K. dggridr: Discrete Global Grids for R. R package version 2.0.4. (2018) doi:10.5281/ZENODO.1322866.
34. Xu, W.-B. *et al.* Regional occupancy increases for widespread species but decreases for narrowly distributed species in metacommunity time series. (2023) doi:10.5281/zenodo.7675355.
35. Blowes, S. A. *et al.* Synthesis reveals biotic homogenisation and differentiation are both common. 2022.07.05.498812 Preprint at https://doi.org/10.1101/2022.07.05.498812 (2022).
36. Stuart, M., Forrest, D., Batt, R., Pinsky, M. & Allen, M. pinskylab/OceanAdapt: Update 2021.1 (v2021.1.0). (2022) doi:10.5281/zenodo.6085448.
